# Massage and Movement Treatment

**Published:** 1888-10

**Authors:** 


### MASSAGE AND MOVEMENT TREATMENT.

A late writer says that Dr. Mitchell does not claim his system of manipulation to be the true plan of massage, but says that massage is a scientific system of physical management of certain diseases, and requires skilled and practiced hands to apply it properly and effectively. It consists of a process of kneading, pressing, stroking rubbihg, hacking and percussion when applied to muscles for the propulsion of blood, lymph and exudations from the periphery toward the center.

Another plan of physical treatment has been lately introduced into this country termed "The Swedish Movement" plan. This is practiced in order to strengthen weak muscles in cases of convalescence and general nervous debility.

Dr. Ling, a Swedish physician, many years ago instituted this plan of treatment. He gave it the name of the "movement plan," which he divided into active and passive. Active movements are such as the patient performs by voluntary muscular contractions, and passive movements such as the patient takes no part in, beyond allowing the operator to move the whole or part of the body—as flexion, extension and rotation, and to manipulate it.

These simple movements Ling combines into resistive, or duplex movements, viz: Active passive, or concentric duplex movements, such as the operator resists; and passive active duplex movements, such as the operator overcomes when the patient resists. Duplex, because two individuals engage in it; concentric because the patient's muscles have to overcome a resistance toward the trunk; eccentric, because the force acts in a direction away from the body.

No doubt, in many instances these plans of treatment might be advantageously supplemented with electricity.

